# Loci on chromosomes 1A and 2A affect resistance to tan (yellow) spot in wheat populations not segregating for *tsn1*

**DOI:** 10.1007/s00122-017-2981-6

**Published:** 2017-09-14

**Authors:** Manisha Shankar, Dorthe Jorgensen, Julian Taylor, Ken J. Chalmers, Rebecca Fox, Grant J. Hollaway, Stephen M. Neate, Mark S. McLean, Elysia Vassos, Hossein Golzar, Robert Loughman, Diane E. Mather

**Affiliations:** 1 Department of Primary Industries and Regional Development (DPIRD), 3 Baron Hay Ct, South Perth, WA 6151 Australia; 2 School of Agriculture and Environment, University of Western Australia, 35 Stirling Hwy, Crawley, WA 6009 Australia; 3 School of Agriculture, Food and Wine, Waite Research Institute, University of Adelaide (UA), Glen Osmond, SA 5064 Australia; 4 Agriculture Victoria, Private Bag 260, Horsham, VIC 3401 Australia; 5 Centre for Crop Health, University of Southern Queensland, Toowoomba, QLD 4350 Australia

## Abstract

*****Key message***:**

**QTL for tan spot resistance were mapped on wheat chromosomes 1A and 2A. Lines were developed with resistance alleles at these loci and at the** ***tsn1*** **locus on chromosome 5B. These lines expressed significantly higher resistance than the parent with** ***tsn1*** **only.**

**Abstract:**

Tan spot (syn. yellow spot and yellow leaf spot) caused by *Pyrenophora tritici*-*repentis* is an important foliar disease of wheat in Australia. Few resistance genes have been mapped in Australian germplasm and only one, known as *tsn1* located on chromosome 5B, is known in Australian breeding programs. This gene confers insensitivity to the fungal effector ToxA. The main aim of this study was to map novel resistance loci in two populations: Calingiri/Wyalkatchem, which is fixed for the ToxA-insensitivity allele *tsn1*, and IGW2574/Annuello, which is fixed for the ToxA-sensitivity allele *Tsn1*. A second aim was to combine new loci with *tsn1* to develop lines with improved resistance. Tan spot severity was evaluated at various growth stages and in multiple environments. Symptom severity traits exhibited quantitative variation. The most significant quantitative trait loci (QTL) were detected on chromosomes 2A and 1A. The QTL on 2A explained up to 29.2% of the genotypic variation in the Calingiri/Wyalkatchem population with the resistance allele contributed by Wyalkatchem. The QTL on 1A explained up to 28.1% of the genotypic variation in the IGW2574/Annuello population with the resistance allele contributed by Annuello. The resistance alleles at both QTL were successfully combined with *tsn1* to develop lines that express significantly better resistance at both seedling and adult plant stages than Calingiri which has *tsn1* only.

**Electronic supplementary material:**

The online version of this article (doi:10.1007/s00122-017-2981-6) contains supplementary material, which is available to authorized users.

## Introduction

Tan spot (syn. yellow spot and yellow leaf spot) caused by the fungus *Pyrenophora tritici*-*repentis* (Died.) Drechs. [anamorph *Drechslera tritici*-*repentis* (Died.) Shoem.] is an important foliar disease of bread wheat (*Triticum aestivum* L.) and durum wheat (*T. turgidum* L. var. *durum*). It was first identified in the United States in the 1940s, and since then has increased in incidence and severity worldwide (Wolf et al. 1998; Ciuffetti and Tuori 1999). Severe epidemics have been reported in Australia (Rees and Platz 1983), Brazil (Mehta and Gaudencio 1991), Europe (Cook and Yarham 1989), and the United States (Schilder and Bergstorm 1989). Implementation of new farm management practices such as minimum tillage, stubble retention, increased intensity of wheat within the cropping system and the cultivation of susceptible varieties have supported the increased prominence of this disease (Rees and Platz 1979; Adee and Pfender 1989).

Wheat can be infected by *P. tritici*-*repentis* at any developmental stage, with disease perpetuation during crop maturity and grain filling having the greatest impact on grain quality and yield (Bankina and Priekule 2011). Planting wheat into infected wheat residues exposes juvenile plants to disease pressure in most regions. Where wet conditions are present during grain filling, the disease can progress to severe levels (Ronis and Semaskiene 2006) and cause yield losses between 20 and 50% as observed in Queensland and Western Australia (Shipton 1968; Rees et al. 1982; Rees and Platz 1983; Bhathal et al. 2003).

Development and adoption of resistant varieties are regarded as the most economically effective, comprehensive and environmentally safe means of controlling tan spot. Resistance to tan spot can be both qualitative (Lamari and Bernier 1989; Gamba and Lamari 1998) and quantitative (Elias et al. 1989; Friesen and Faris 2004). Genes such as *tsn1, tsn2, tsn3, tsn4, tsn5, tsn6*, *tsn*-*syn1*, and *Tsn*-*syn2* are known to affect toxin sensitivity and to confer race-specific resistance to necrosis (Anderson et al. 1999; Singh et al. 2006, 2008; Tadesse et al. 2006a, b, 2010), while the recessive genes *tsc1* and *tsc2* confer resistance to chlorosis (Effertz et al. 2002; Friesen and Faris 2004; Abeysekara et al. 2010). Quantitative trait loci (QTL) have been mapped on almost all chromosomes of wheat (Faris and Friesen 2005; Chu et al. 2008, 2010; Sun et al. 2010; Li et al. 2011; Faris et al. 2012; Singh et al. 2012; Patel et al. 2013). These loci, which were detected after inoculation with mixtures of isolates, are not known to exhibit any race-specific effects. Combining qualitative and quantitative resistance may provide cultivars with high levels of durable resistance.

*P. tritici*-*repentis* is known to produce at least three host-specific toxins (HSTs) that interact with specific host sensitivity genes to cause necrosis and/or extensive chlorosis (Ali et al. 1999; Lamari et al. 1995; Strelkov et al. 2002; Lamari et al. 2005). The Ptr ToxA causes necrosis and sensitivity is conditioned by the gene *Tsn1* on chromosome arm 5BL (Faris et al. 1996; Anderson et al. 1999). Ptr ToxB and Ptr Tox C cause chlorosis, and sensitivity is conditioned by the *Tsc2* gene on chromosome arm 2BS (Friesen and Faris 2004) and the *Tsc1* gene on chromosome arm 1AS (Effertz et al. 2002), respectively. Isolates of the pathogen can possess combinations of these toxins and can be classified into eight races based on the presence or absence of each of the three toxins. All eight of these combinations have been found among North American isolates. Isolates have also been discovered that lack both ToxA and ToxB, yet are able to induce ToxA-like necrotic symptoms on some cultivars of wheat (Ali et al. 2010). These isolates may contain one or more uncharacterised toxins.

In a survey of 119 isolates of *P. tritici*-*repentis* collected in Australia, Antoni et al. (2010) detected the gene for ToxA in all isolates, but did not detect the gene for ToxB. The absence of ToxB in Australian isolates is useful information for guiding the choice of resistance genes for use in wheat breeding. The presence of ToxC has been detected in some Australian isolates (CS Moffat, personal communication, 9 June, 2017).

Consistent with the prevalence of ToxA among Australian isolates of the pathogen, *tsn1* is considered as an important resistance gene in Australian wheat breeding. Selection for *tsn1* can be achieved with molecular markers or with phenotypic screens for ToxA insensitivity (Faris et al. 1996). However, this gene does not explain the full spectrum of resistance in Australian wheat germplasm. Further, Faris et al. (2012) showed that the amount of variation explained by *tsn1* can vary considerably among different isolates and suggested that regulation of the ToxA gene may vary amongst isolates.

Identification of resistance factors other than *tsn1* could lead to enhancement of tan spot resistance in wheat. Such factors will be most readily detected in materials that are not segregating for the *Tsn1* and *tsn1* alleles. The aims of this study were to map loci for tan spot resistance in two wheat populations: one fixed for the ToxA-insensitivity allele *tsn1* and the other fixed for the ToxA-sensitivity allele *Tsn1* and to combine these new loci with *tsn1* to develop lines with improved resistance.

## Materials and methods

### Plant materials

Two populations of doubled haploid (DH) lines were provided by InterGrain Pty Ltd (Perth, Western Australia, Australia). The populations had been developed using wheat × maize wide-crossing system at the Department of Primary Industries and Regional Development (DPIRD). One population (designated 05Y002) consisted of a random sample of 247 lines derived from the F_1_ generation of a cross between the cultivars Calingiri and Wyalkatchem, both of which carry the *tsn1* gene for insensitivity to ToxA. The other population (designated 03Y260) consisted of a random sample of 97 lines from a cross between a breeding line, IGW2574 and Annuello, neither of which carries *tsn1*. Wheat cultivars ranging in their tan spot resistance were used as controls. These included H45 [moderately resistant (MR)], Magenta (MR), Cunderdin [moderately resistant to moderately susceptible (MRMS)], EGA Bonnie Rock (MRMS), Mace (MRMS), Arrino [moderately susceptible to susceptible (MSS)], Reeves (MSS), Machete [susceptible (S)], Correll [susceptible to very susceptible (SVS)], Datatine (SVS), Yitpi (SVS) and Gutha [very susceptible (VS)].

### Genetic map development

DNA was isolated from leaf tissue sampled from one seedling per line using a mini-prep ball bearing extraction method (Rogowsky et al. 1991) with some modifications (Pallotta et al. 2000). DNA from each line was assayed on a DArT marker array (Akbari et al. 2006) by Diversity Arrays Technology Pty Ltd (Canberra, ACT, Australia), with each DArT marker scored based on the presence or absence of hybridisation. Simple-sequence repeat (SSR) markers were assayed using Multiplex-Ready technology (Hayden et al. 2008). Several single-nucleotide polymorphisms (SNPs) were assayed using KASP technology (LGC Limited, Teddington, Middlesex, UK).

Within each population, a Chi-squared test was used to compare the observed segregation ratio of each marker to the expected ratio of 1:1. Markers showing significant segregation distortion (*p* < 0.01) or markers for which data were available for fewer than 80% of the lines were excluded from map construction. The remaining markers were grouped into linkage groups and initially ordered within linkage groups using the program Multipoint (http://www.multiqtl.com/). Marker order was obtained using the MSTmap (Wu et al. 2008) function in the ASMap package (Taylor and Butler 2017) available in the R statistical computing environment (R Core Team 2016). Genetic distances between marker loci were determined using the hidden Markov algorithm derived by Lander and Green (1987). Linkage groups were assigned to chromosomes and oriented within chromosomes via comparisons with published maps. Final linkage maps are presented in Supplementary Material 1. Missing allele scores were imputed using the flanking marker algorithm of Martínez and Curnow (1992).

Based on the results of QTL mapping, one chromosome from each cross (1A for IGW2574/Annuello and 2A for Calingiri/Wyalkatchem) was selected for more detailed analysis. Additional SNPs were assayed on these chromosomes. For some SNPs, KASP primer sequences (Supplementary Material 2) were obtained from CerealsDB (http://cerealsdb.uk.net; Wilkinson et al. 2012). For others, KASP primers were designed from new sequences. Some of these sequences were kindly provided by Dr. Timothy March of the University of Adelaide. Others were obtained via application of DArTseq technology (Diversity Arrays Technology Pty Ltd) to the mapping parents and selected lines. KASP markers were designed and assayed using software (Kraken), instruments (a SNPLine system) and reagents from LGC Limited. For each of the two chromosomes, a new linkage map was constructed using only the KASP marker data.

### Phenotypic evaluation

The two DH populations, their parents and the control lines were phenotyped at South Perth, Western Australia (S31°59.20′, E115°53.13′) during 2009, 2010, and 2011; at Horsham, Victoria (S36°74.56′, E142°11.13′) during 2010 and at Toowoomba, Queensland (S27° 32.00′, E151° 56.15′) during 2011, 2012, and 2013.

#### South Perth, Western Australia

Inoculum was prepared using a modified method of Evans et al. (1993). Five 4 mm^2^ plugs of isolate WAC11137 (obtained from WAC, Plant Pathology Culture Collection, Perth, Western Australia) were excised from the periphery of a freshly growing culture on potato dextrose agar and transferred to 200 ml of potato dextrose broth (PDB) in a 500-ml Erlenmeyer flask. Flasks were incubated at room temperature and light conditions on an orbital shaker at 250 rpm for 5 days. The mycelial mat was poured onto a sterile nylon screen and squeezed with a sterile spatula to remove the excess PDB. A sample of 10 g of the mycelial mass was homogenised in 20 ml of sterile distilled water for 30 s at 13,500 rpm using a TH-220 tissue homogenizer (Omni International, Marietta, GA, USA) with a G10-195 saw tooth probe. A sample of 20 ml of the homogenised mycelial suspension was added to 1 l of liquid clarified V8 juice agar medium maintained at 48 °C. Flasks were swirled to distribute the homogenised mycelium in the liquid agar medium and poured into Petri plates. Plates were incubated for 48 h at 24 °C in darkness after which aerial mycelium was appressed to the agar surface using three drops of sterile distilled water and a sterile bent rod. Plates were then incubated for 24 h at 24 °C under a combination of fluorescent and near-ultraviolet light (50 μmol/m^2^/s) to induce conidiophore production and then for 24 h at 16 °C under darkness to induce conidial production. Plates were examined under a dissection microscope to verify conidial production and then stored in air tight plastic bags at −20 °C, with spores remaining viable for up to 1 year. For inoculation, plates were thawed at room temperature for 1 h and spores harvested by spinning agar discs in 0.5% gelatine solution (e.g., 50 discs in 500 ml of gelatine solution) at 175 rpm for 5 min in a 4-l glass beaker. Approximately 80,000–130,000 spores per Petri plate were produced using this method. The spore concentration was adjusted to 3000 spores/ml in 0.5% gelatine solution for all inoculations.

During 2009, both populations, their parents and the control lines were evaluated for resistance in an irrigated field nursery in a randomised block design with three replications. The three replicate blocks of the experiment were sown at one-week intervals, in the second, third and fourth weeks of May. Each experimental unit consisted of a 30-cm row in which 15 seeds were sown. Rows were 20 cm apart. Plots were fertilised with a mixture of superphosphate, urea and potash (6:4:1) at a rate of 100 kg/ha at planting and at 8 weeks after sowing. Plots were protected from powdery mildew infection with 250 g/ha of Quinoxyfen and 125 g/ha Bupirimate applied at 4-week intervals for 12 weeks. Blanket spray inoculations using conidial suspensions were done for each block when most lines had started to tiller (Zadoks growth stage (Z) 21; Zadoks et al. 1974) and again at half head emergence (when most lines had reached Z55). To promote humidity for fungal infection, plots were watered prior to inoculation. After inoculation, blocks of plots were covered by plastic tents and shade cloth (84–90% cover factor) for 48 h. Seven days after the first (tillering-stage) inoculation, disease severity was assessed for each plot using a 0–5 scale where 0 is no disease and 5 is high disease. Two weeks after the second (heading-stage) inoculation, percentage leaf area diseased (PLAD) was assessed on the flag leaf and the leaf below (flag-1) of five plants in each plot. Mean PLAD values were calculated for each plot and used for analysis. The Zadoks growth stage of each plot was recorded 12 weeks after sowing.

In 2010, a similar experiment was conducted, but with modifications to the trial design and inoculation protocol in order to overcome possible effects of plant maturity and height on disease expression at the adult plant stage (Shankar et al. 2008). Lines were sown in plots consisting of two 10-cm rows 10 cm apart, with up to ten seeds sown per row and with 30 cm between adjacent plots. Plots were fertilised and protected from powdery mildew infection as described above. Individual plots were inoculated at different times as they reached heading (Z55), by spraying flag leaves with the conidial suspension to run-off. High humidity was ensured by watering the site just before inoculation and by using plastic bags secured over PVC rings (15 cm high, 30 cm diameter) to cover individual plots for 48 h after inoculation (Shankar et al. 2008). Before being used to cover the plots, the plastic bags were misted internally with water. To shade the inoculated plants from direct sunlight, the plastic bags themselves were covered with shade-cloth bags (84–90% cover factor). At 390 °C thermal days (sum of mean daily temperatures) after inoculation, PLAD was scored on the flag leaves of five individual plants in each plot and a mean was calculated and used for analysis.

For phenotypic evaluation of seedlings, glasshouse experiments were conducted during both 2009 and 2010 with both populations, their parents and the control lines. Lines were sown in 120-mm-diameter pots containing a sand-loam mix with 1 g of Osmocote (slow release fertiliser). The experiments were conducted in three-replicate randomised block designs, with four lines sown within each pot. Temperatures were maintained at 24 °C during the day and 22 °C at night. At Z12.5, seedlings were spray inoculated to run-off with the conidial suspension as described above. Inoculated plants were incubated for 24 h in a humidifier. Nine days after inoculation, a 0–5 scale was used to rate disease symptoms on the leaves that had been fully emerged at inoculation. In 2010, an additional experiment was conducted in which the two populations were inoculated at tillering (Z22) and rated 9 days later.

In 2011, the IGW2574/Annuello population, its parents and the control lines were grown in a controlled environment with 24/22 °C day/night temperatures. The experiment was conducted in a randomised block design with three replicates. Four seeds per line were sown within each pot. Growing medium, fertiliser, seedling inoculation and the initial disease rating were the same as described above for the glasshouse experiments. Immediately after the initial disease rating, plants were provided with a 20-h photoperiod consisting of 12 h of natural day light and 8 h of high pressure sodium light with an active radiation of 400–500 μmol/m^2^/s. Plants were fertilised with soluble all purpose Thrive N:P:K 25:5:8.8 (Yates Australia, Padstow NSW) at a concentration of 0.8 g/l and a rate of 60 ml/pot on a weekly basis and with a trace element solution of Liberal BMX (BASF) at a concentration of 0.5 g/l and a rate of 30 ml/pot on a fortnightly basis. At heading (Z55), flag leaves of individual pots were inoculated as described above. Fourteen days after this inoculation, disease severity symptoms were rated on a percentage scale.

#### Horsham, Victoria

In the field, during 2010 three replicates of each line of the two populations, parents and controls were sown in randomised block design in an irrigation bay on 18 May 2010. Each experimental unit consisted of a 50-cm row in which approximately 20 seeds were sown. Rows were 30 cm apart. One week prior to sowing the field site was pre-drilled with mono-ammonium phosphate fertiliser (70 kg/ha) treated with flutriafol fungicide (75 g/ha) to suppress stripe rust (*Puccinia striiformis*) development. Infection was established by spreading approximately 1 t/ha of wheat stubble naturally infected with locally occurring *P. tritici*-*repentis* from the previous year in June 2010. The site was flood irrigated twice during season to stimulate disease development. Disease severity was rated on a 1–9 scale where 1 is low disease severity and 9 is high disease severity at tillering (Z22) and booting (Z45).

For glasshouse experiments, inoculum was prepared using methods described by Mclean et al. (2010) for *Pyrenophora teres* f. *maculata*. Three virulent isolates 07-0003, 03-0025, 03-0152 (obtained from the culture collection of Agriculture Victoria, Horsham) were grown on potato dextrose agar under white fluorescent and growlux lights at 20 °C for 5–7 days. Two 4 mm^2^ plugs of each cultured isolate were then sub-cultured onto V8 juice agar plates and incubated in the conditions described above for 6 days. Inoculum was prepared by scraping conidia and mycelium from the surface of the plates using an electric toothbrush. Two seeds of each line were sown into 5-cm pots containing potting mixture, fertiliser and trace elements. Lines were arranged in a four-replicate randomised complete block design under natural light at 20 ± 2 ºC. Seedlings were inoculated at the two-leaf stage (Z12) with a spore and mycelium suspension with concentration of ~80,000 parts per ml of spores and mycelial fragments. Inoculated plants were incubated at 95–100% humidity at a temperature of 20 ± 1 ºC for 72 h with the first 24 h in darkness and the following 48 h under a 12-h photoperiod. Inoculated seedlings were then returned to a glasshouse for 8 days to allow symptom development, after which the seedlings were assessed for symptom severity using a 1–9 scale.

#### Toowoomba, Queensland

During 2011, both populations, their parents and the control lines were sown into a field nursery in a two-replicate randomised block design under shade cloth. To enhance pathogen sporulation and infection, humidity was increased two or three times weekly, as necessary, at sunset using rainwater supplied micro misters for 30–60 min. Each plot was a 20-cm-long single row with 20 cm between plots. To avoid drought stress, plants were irrigated approximately fortnightly using drip irrigation. Infection was established by spreading between rows, infected stubble from the previous year. At early tillering (Z22), tan spot symptom severity was rated on a 1–9 scale.

During 2012–2013, both populations, their parents and the control lines were assessed in a controlled environment room at 23 ± 1 °C with each lighting fixture containing both Sodium Vapour and Metal Halide bulbs emitting PAR at 400–500 μmol/m^2^/s. Prior to seedling rating plants were grown with 14-h day and 10-h night and post rating were switched to 20-h day and 4-h night to reduce the time to head emergence. Plants were grown at 20–22 °C in a 14-h day in 55-mm Square Native Tube pots, 160 mm high (Garden City Plastics, Monbulk, VIC, Australia), containing Searles Native Mix potting soil (Searles Pty Ltd, Kilcoy, QLD, Australia). Seeds were pre-germinated at 5 °C and four seeds per entry were planted in each pot. The statistical layout was a partially replicated design. For all of the experiments conducted in Queensland, an isolate BRIP28204 originally isolated from wheat in the Darling Downs QLD in 1999 was used (Queensland Plant Pathology Herbarium, BRIP, Brisbane, Australia). The isolate, sub-cultured a maximum of three times after isolation from infected leaves, was grown on thick plates of clarified V8 juice agar medium (approx. 15 ml per 9 cm diameter Petri plate) and incubated for 5 days in the dark at 25 °C after which aerial mycelium was appressed to the agar surface using three drops of sterile distilled water and a sterile bent rod. Plates were then incubated at 22 °C for 8 h under a combination of fluorescent and near UV light to induce conidiophore production and then for 16 h under darkness to induce conidial production. Sporulating plates were stored at −70 °C for up to 1 year. For inoculation, sporulating plates were thawed at room temperature and spores harvested by applying a strong spray of deionized H_2_O to the culture surface to dislodge spores. One drop of tween was used per 100 ml of spore suspension and the suspension was kept on ice and with constant agitation until inoculation. For both adult and seedling screening spore concentration was adjusted to 120 ± 30 spores per ml and at growth stage Z12.5 the top 2–3 seedling leaves were inoculated with 1 ml of spore suspension. Inoculated plants were incubated in the dark for about 36 h with a humidifier operating 30 min on and 30 min off. When supplementary susceptible control plants exhibited expected disease reactions (at around 9 days after inoculation), disease was assessed on a 1–9 scale on the leaves that had been fully emerged at inoculation. Immediately after rating, plants were returned to the growth room and foliar and soil fertilised with half strength soluble Thrive fertiliser (Yates Australia, Padstow NSW), on a weekly basis. When individual plants had reached growth stage Z55, flag leaves of individual pots were inoculated at around Z55 as above with approximately 3 ml of spore suspension and rated 14 days later using a 1–9 scale.

### Statistical methods

#### Single experiment linear mixed model

Tan spot severity measurements recorded as percentages were scaled to proportions and logit transformed to ensure model assumptions were adequately satisfied. The remaining tan spot severity traits (recorded as 0–5 and 1–9 rating scales) and data for developmental traits (Zadoks growth stage, days to heading and plant height) were analysed on their original scales. Each trait was then initially analysed using a linear mixed model that partitions and accounts for genetic and non-genetic information arising from the field, glasshouse or controlled-environment. The linear mixed model had the form:

1 $$\varvec{y} = \varvec{X\beta } + \varvec{Zu} + \varvec{Z}_{\varvec{g}} \varvec{g} + \varvec{e,}$$

where ***y*** is a vector of trait observations and ***Xβ*** is the fixed component of the model containing a term that captures the mean effects of the DH population, parents and control lines. This component may also contain covariate information such as terms to adequately capture linear trends that may exist in the environment of the experiment. The ***Zu*** is a random component consisting of terms used to model non-genetic variation due to the experimental design such as blocks or benches. The component ***Z***_***g***_ ***g*** was used to model the underlying genetic variation of the trait among the DH lines. Where appropriate, the residual error, ***e***, was used to capture extraneous variation or correlation that may arise from known dependencies between observations in an experiment. For example, in the field trials, the error term consisted of a residual variance as well as a correlation structure parameterized as a separable AR1 × AR1 (AR1 = auto-regressive process of order 1) that models the dependency of the observations due to the proximity of neighbouring plots in the experiment. The set of effects (***u***, ***g***, ***e***) were considered to be mutually independent. For each of the fitted trait models, the best linear unbiased predictors (BLUPs) of the DH lines were extracted and broad-sense heritabilities were calculated using the formula derived by Cullis et al. (2006).

#### Multiple experiment linear mixed model

For each of the populations, the complete set of tan spot severity traits collected across the experiments were analysed in a multi-experiment (ME) linear mixed model of the form described in (1). To ensure a uniform trait measurement, tan spot ratings that were taken on a 0–5 or 1–9 scale in any experiment were scaled proportions and then logit transformed. The ME linear mixed model consisted of the complete set of non-genetic terms associated with each of the single experiment models. It also contained a genotype-by-experiment term (Smith et al. 2005) to capture the genetic variation of the DH lines in each experiment as well as the genetic correlation of the DH lines between each pair of experiments. From the fitted ME linear mixed model, the estimated genetic correlation matrix was extracted and summarised.

#### QTL analyses

For all traits in each experiment, the whole genome average interval mapping (WGAIM) approach was used for QTL analysis (Verbyla et al. 2007, 2012). This used an extension of the linear mixed model defined in (1) by incorporating a whole genome approach for detection and selection of QTL. Preceding the analysis, pseudo-intervals were calculated at the mid-points between adjacent markers using Verbyla et al. (2007). A working linear mixed model was then proposed that includes all pseudo-intervals as random covariates with a single variance parameter. This parameter was then tested for significance using a likelihood ratio test of the working model against the initial linear mixed model. If it was significant, an outlier statistic is calculated for every interval and the interval with the largest outlier statistic is selected as a QTL. The interval was then removed from the contiguous block of intervals and placed in the model as a separate random covariate. This forward selection process was repeated until the variance parameter associated with the remaining random pseudo-interval effects was not significant. The additive set of selected QTL was then summarised with their location on the genome, the sizes of the QTL effects, their LOD scores of significance and their percentage contribution to the total genetic variance.

For each of the populations, an ME linear mixed model QTL analysis was conducted using the ordered set of KASP markers spanning the chromosome containing the main QTL of interest. This was achieved through the incorporation of individual markers as a marker-by-interaction fixed component in the ME linear mixed model. Each marker was then considered in turn and a separate ME QTL linear mixed model was fitted for all markers spanning the chromosome (Bonneau et al. 2013). To detect the marker most likely linked to the putative QTL, the marker-by-experiment term for each ME QTL linear mixed model was tested under a null hypothesis that all single marker effects across experiments were simultaneously zero. A Wald statistic (Kenward and Roger 1997) was calculated for each marker and used to form an ME QTL profile spanning the chromosome. The peak of this Wald statistic profile was considered to be the marker most likely linked to the QTL and the KASP markers at this location were then summarised across individual experiments showing their LOD (logarithm base 10 of odds) scores. Linkage maps of chromosomal regions were drawn using MapChart (Voorrips 2002). Additionally, to understand the reduction in disease symptoms achievable when the favourable allele is used with these markers, the marker effect sizes were back-transformed from the logit scale and the average reduction in the severity of tan spot was calculated from a fixed level of 50% disease severity.

#### Computations

All models were analysed using the flexible linear mixed-modelling software ASReml-R (Butler et al. 2009) available in the open-source statistical software platform R (R Core Team 2016). For all single experiment QTL analyses, the R package wgaim (Taylor et al. 2011; available at the Comprehensive R Archive Network, http://CRAN.R-project.org/package=wgaim) was used. The package uses the linear mixed model functions in ASReml-R for fitting QTL models and also contains tools for post model diagnostics and summary of the QTL. For the multi-experiment QTL analysis, unpackaged R code was used to implement the marker scanning algorithm.

### Development and evaluation of lines with resistance alleles at three loci

Marker-assisted single-seed descent was used to develop lines that carry alleles at two QTL with *tsn1*. One line was selected from each population based on good resistance and the presence of resistance-associated marker alleles in the QTL region (on 1A in IGW2547/Annuello and on 2A in Calingiri/Wyalkatchem). These lines were crossed with each other. All F_1_s were ‘grass clumps’. These were grown at a constant 26 °C temperature and treated with 0.5 g/l of gibberellic acid at 2-day intervals (Yao and Canvin 1969) to induce flowering. One hundred and ninety-four F_2_ seeds were obtained. Progeny were progressed through subsequent generations by single seed descent with marker-assisted selection applied for the resistance allele of each QTL, for the *tsn1* allele and for homozygosity at the *Vrn*-*A1* phenology locus. Sixteen F_5_ lines homozygous for the resistance alleles at the three tan spot resistance loci and at *Vrn*-*A1* were selected. These lines, along with the four original parents and a larger set of lines (not discussed in this paper) were assessed at the seedling and adult stages in a controlled environment and at the adult stage in the field at South Perth. At the seedling stage, a rating scale of 0–5 was used. At the adult stage, the percentage of diseased flag leaf was estimated.

Tan spot severity measurements recorded as percentages were logit transformed and all traits were analysed using the single experiment linear mixed model modelling method described above. In this validation model, the grandparents of the two populations, the parents selected for this crossing scheme and the 16 derived F_5_ lines were fitted as fixed effects to ensure accurate comparative hypothesis testing of their means could be achieved. The remaining lines were fitted as random effects to adequately capture their genetic variation. For each trait, the best linear unbiased estimator (BLUE) for each line was extracted from the fitted model. A least significant difference (LSD) was then calculated at an alpha level of 0.05 and used to broadly compare BLUEs. For percentage traits, all inference was performed on the scale of the transformation. For these traits, the extracted BLUEs were back-transformed to simplify interpretation.

## Results

### Phenotypic analysis

Estimated broad-sense heritability (*H* ^2^) for disease severity was high (0.70–0.95) in most experiments but lower in some experiments conducted at Toowoomba (Table 1). In the field experiments conducted at South Perth, heritability estimates were very high for Zadoks growth stage assessed at 12 weeks (2009: 0.93 for Calingiri/Wyalkatchem and 0.89 for IGW2574/Annuello) and days to heading (2010: 0.97 in Calingiri/Wyalkatchem and 0.91 in IGW2574/Annuello).

**Table 1:** Tan spot severity traits recorded in experiments in which Calingiri/Wyalkatchem (C/W) and IGW2574/Annuello (I/A) wheat populations were evaluated

Location	Year	Environment type	Trait	Stage(s) of inoculation	Stage of symptom assessment	Type of symptom assessment	Heritability estimate	
							C/W	I/A
South Perth	2009	Glasshouse	Tan spot severity (seedling)	Seedling	9 days after inoculation	Rating (0–5)	0.79	0.81
		Field	Tan spot severity (tillering)	Tillering	7 days after inoculation	Rating (0–5)	0.73	0.71
			Tan spot severity (adult)	Tillering, heading	14 days after second inoculation	Percentage	0.86	0.92
	2010	Glasshouse	Tan spot severity (seedling)	Seedling	9 days after inoculation	Rating (0–5)	0.82	0.84
			Tan spot severity (tillering)	Tillering	7 days after inoculation	Rating (0–5)	0.81	0.82
		Field	Tan spot severity (adult)	Heading	390 °C thermal days after inoculation	Percentage	0.78	0.87
	2011	Controlled	Tan spot severity (seedling)	Seedling	Seedling	Rating (0–5)	–	0.95
			Tan spot severity (adult)	Seedling, heading	9 days after second inoculation	Percentage	–	0.95
Horsham	2010	Glasshouse	Tan spot severity (seedling)	Seeding	Seedling	Rating (1–9)	0.70	0.79
		Field	Tan spot severity (tillering)	Seedling	Tillering	Rating (1–9)	0.77	0.71
			Tan spot severity (booting)	Seedling	Booting	Rating (1–9)	0.70	0.64
Toowoomba	2011	Field	Tan spot severity (tillering)	Seedling	Tillering	Rating (1–9)	0.58	0.66
	2012	Controlled	Tan spot severity (seedling)	Seedling	Seedling	Rating (1–9)	0.53	0.49
	2013	Controlled	Tan spot severity (seedling)	Seedling	Seedling	Rating (1–9)	0.73	0.55
			Tan spot severity (adult)	Seedling, heading	14 days after second inoculation	Rating (1–9)	0.46	0.81

**Fig. 1:**
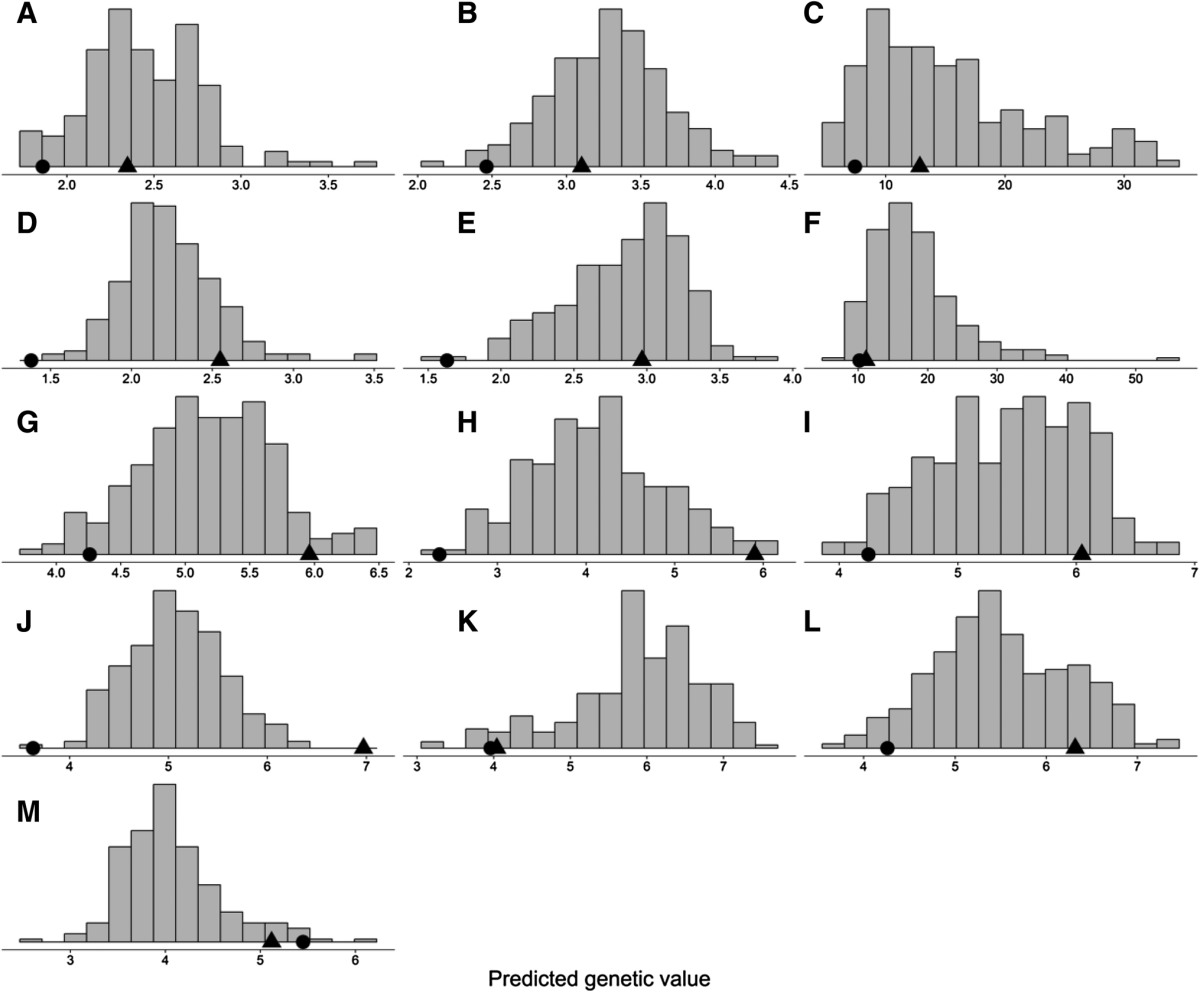
Genetic distribution of tan spot severity in the doubled haploid population Calingiri/Wyalkatchem for South Perth 2009 Glasshouse (**a** seedling), South Perth 2009 Field (**b** tillering; **c** adult), South Perth 2010 Glasshouse (**d** seedling, **e** tillering), South Perth 2010 Field (**f** adult), Horsham 2010 Glasshouse (**g** seedling), Horsham 2010 Field (**h** tillering, **i** booting), Toowoomba 2011 Field (**j** tillering), Toowoomba 2012 controlled environment (**k** seedling) and Toowoomba 2013 controlled environment (**l** seedling, **m** flag leaf). The relative position of the parents is indicated in each plot (Calingiri = filled triangles; Wyalkatchem = filled circles)

**Fig. 2:**
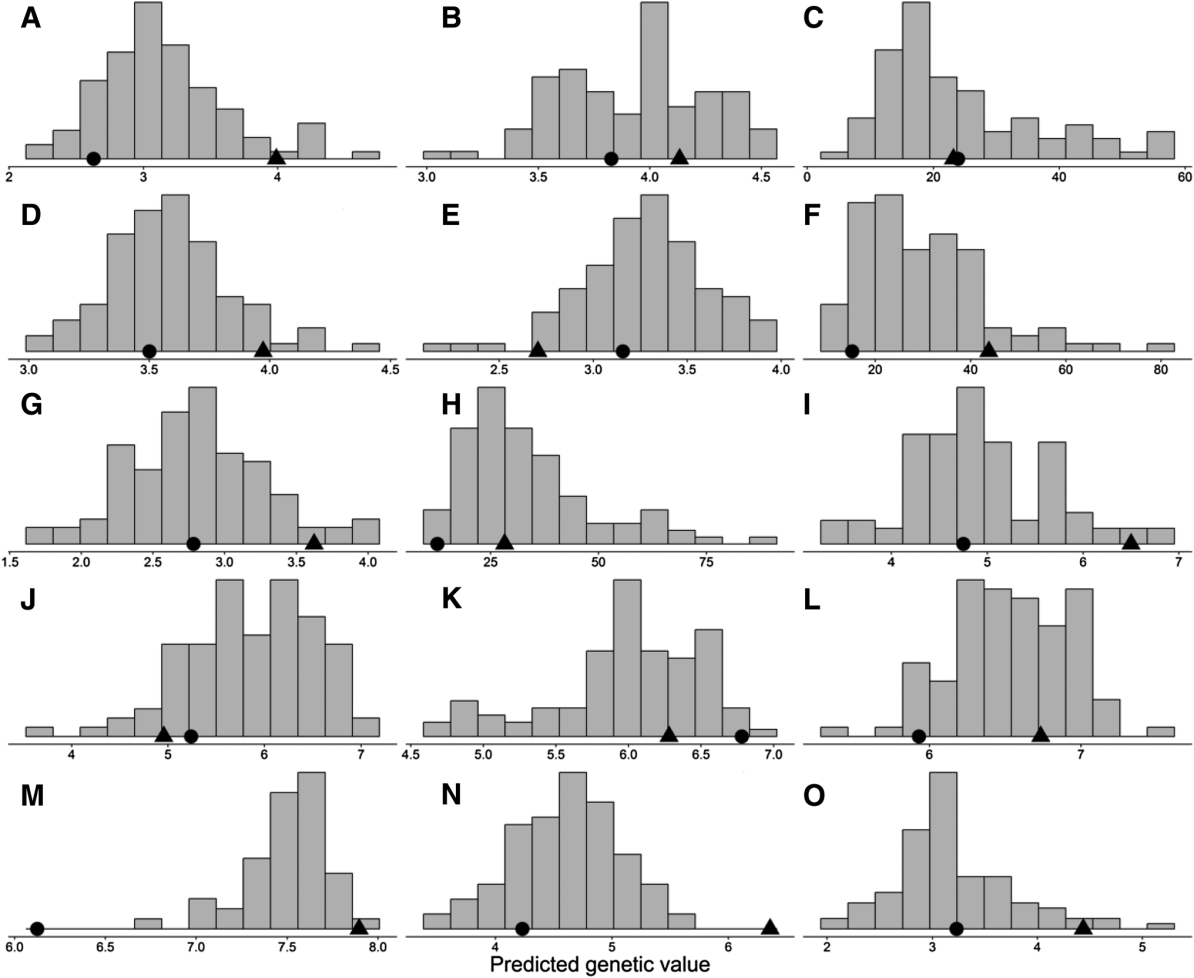
Genetic distribution of tan spot severity in the doubled haploid population IGW2574/Annuello for South Perth 2009 Glasshouse (**a** seedling), South Perth 2009 Field (**b** tillering, **c** adult), South Perth 2010 Glasshouse (**d** seedling, **e** tillering), South Perth 2010 Field (**f** adult), South Perth 2011 controlled environment (**g** seedling, **h** adult) Horsham 2010 Glasshouse (**i** seedling), Horsham 2010 Field (**j** tillering, **k** booting), Toowoomba 2011 Field (**l** seedling), Toowoomba 2012 controlled environment (**m** seedling) and Toowoomba 2013 controlled environment (**n** seedling, **o** adult). The relative position of the parents is indicated in each plot. (IGW2574 = filled triangles; Annuello = filled circles)

In most cases, tan spot was more severe on Calingiri compared to Wyalkatchem in the Calingiri/Wyalkatchem population. Similarly, in most cases, tan spot severity was greater on IGW2574 compared to Annuello in the IGW2574/Annuello population. DH lines of both populations exhibited continuous distributions and transgressive segregation for tan spot severity (Figs. 1, 2).

In the field experiments conducted at South Perth, phenotypic correlation between years was strong for the developmental traits (Zadoks growth stages in 2009 and days to heading in 2010) (*r* = −0.89 for Calingiri/Wyalkatchem and *r* = −0.83 for IGW2574/Annuello). Tan spot severity on adult plants was strongly correlated with the growth stages recorded in 2009 (*r* = 0.70 for Calingiri/Wyalkatchem and 0.63 for IGW2574/Annuello), but not with the numbers of days to heading recorded in 2010 (*r* = −0.09 Calingiri/Wyalkatchem and −0.001 for IGW2574/Annuello). This indicates that confounding effects of differential maturity on disease expression were successfully removed in 2010 by modification of the experimental process which included inoculating individual plots at a specific growth stage and rating at a specific thermal time after inoculation rather than inoculating all plots in a replicate on the same day and rating 2 weeks after inoculation.

Estimated genetic correlations between disease scores at various growth stages and experiments were extracted from the fitted ME linear mixed model for each population (Supplementary Material 3). The broad range of estimated genetic correlations, *r* = −0.22 to 0.95, indicate the potential presence of genotype-by-experiment interactions and the possibility of different genes controlling resistance at various growth stages. For the Calingiri/Wyalkatchem population, tan spot severity in field experiments across different sites were moderate to strongly genetically correlated (*r* = 0.30–0.75). With the exception of the controlled environment of Toowoomba 2013, tan spot severity of field experiments at any given location correlated well with tan spot severity in glasshouse and controlled environment experiments at the same location. This indicates that it might be possible to replace field experiments with rapid experiments that could be conducted at any time of year in the glasshouse and controlled environment. For the IGW2574/Annuello population, weak to strong genetic correlations (*r* = 0.16–0.67) were detected for tan spot severity between field experiments. Tan spot severity was also strongly correlated (*r* = 0.70) between the 2010 South Perth field experiment and 2011 South Perth controlled environment. The 2012 Toowoomba glasshouse experiment had poor genetic correlation with the other experiments (*r* = −0.22 to 0.32).

### QTL analysis: Calingiri/Wyalkatchem

In the Calingiri/Wyalkatchem population, a QTL was detected on chromosome 2A for tan spot severity, with the Wyalkatchem parental allele favouring resistance (Table 2). In most cases this QTL was estimated to be in the 7.6-cM interval between markers *rPt*-*9057* and *tPt*-*8937*, but in two experiments the position estimates were in other nearby intervals. The only other QTL region that was detected in more than one experiment was one on chromosome 6B that affected tan spot severity at tillering and on adult plants in the 2010 field experiment at South Perth and at tillering in the 2010 experiment at Horsham. In addition, QTL were detected on chromosomes 1A, 2D, 3A, 3D and 7B, but none of these were detected in more than one environment.

**Table 2:** Quantitative trait loci detected in the Calingiri/Wyalkatchem population

Chromosome (linkage group)	Flanking markers (cM positions)	Trait	Location	Year	Environment type	*p*	% Variance	LOD	Source of allele that reduces trait value
1A (1)	*wPt*-*7296* (0)–*wPt*-*6280* (1.3)	Tan spot severity (adult)	South Perth	2009	Field	0.002	3.6	1.7	Calingiri
2A (1)	*wPt*-*9320* (22.7)–*BS00011865* (26.1)	Tan spot severity (seedling)	Toowoomba	2013	Controlled	0.000	33.5	10.3	Wyalkatchem
	*iwa5893* (32.5)–*cfa2263* (37.5)	Tan spot severity (booting)	Horsham	2010	Field	0.000	19.3	6.2	Wyalkatchem
	*rPt*-*9057* (38.7)–*tPt*-*8937* (46.4)	Tan spot severity (seedling)	South Perth	2009	Glasshouse	0.000	10.3	2.7	Wyalkatchem
		Tan spot severity (seedling)	South Perth	2010	Glasshouse	0.000	9.2	2.5	Wyalkatchem
		Tan spot severity (seedling)	Horsham	2010	Glasshouse	0.000	27.4	8.6	Wyalkatchem
		Tan spot severity (tillering)	South Perth	2009	Field	0.000	16.2	4.5	Wyalkatchem
		Tan spot severity (tillering)	Horsham`	2010	Field	0.000	25.6	8.7	Wyalkatchem
		Tan spot severity (adult)	South Perth	2010	Field	0.000	15.1	4.1	Wyalkatchem
	*tPt*-*8937* (46.4)–*wri65* (48.3)	Tan spot severity (adult)	South Perth	2009	Field	0.000	27.4	11.4	Wyalkatchem
	*wri66* (48.3)–*wri64* (49.5)	Tan spot severity (tillering)	Toowoomba	2011	Field	0.000	14.8	5.2	Wyalkatchem
2B (2)	*wPt*-*7004* (94.8)–*wPt*-*1394* (108.1)	Tan spot severity (adult)	South Perth	2009	Field	0.003	3.7	1.7	Calingiri
	*wPt*-*6471* (9.2)–*wPt*-*8548* (11.7)	Tan spot severity (seedling)	Toowoomba	2012	Controlled	0.000	14.2	2.9	Calingiri
2D (1)	*wPt*-*4242* (113.6)–*wPt*-*4413* (123.4)	Tan spot severity (adult)	South Perth	2009	Field	0.004	3.8	1.6	Wyalkatchem
3A (1)	*tPt*-*1143* (0)–*wPt*-*3839* (4.6)	Tan spot severity (booting)	Horsham	2010	Field	0.001	5.6	2.1	Wyalkatchem
3D (1)	*wPt*-*10018* (0)–*wPt*-*733412* (22.1)	Tan spot severity (booting)	Horsham	2010	Field	0.000	6.4	2.4	Wyalkatchem
6B	*wPt*-*6208* (83.4)–*wPt*-*729979* (84.9)	Tan spot severity (tillering)	Horsham	2010	Field	0.000	10	3.8	Wyalkatchem
	*tPt*-*1723* (87.6)–*wPt*-*9195* (88.3)	Tan spot severity (adult)	South Perth	2009	Field	0.001	4.2	1.9	Wyalkatchem
	*wPt*-*0446* (96.6)–*wPt*-*8183* (99.2)	Tan spot severity (tillering)	South Perth	2009	Field	0.000	8.3	2.4	Wyalkatchem
7B (1)	*wPt*-*7046* (78.9)–*wPt*-*744987* (80.2)	Tan spot severity (booting)	Horsham	2010	Field	0.000	9.9	3.5	Calingiri

**Fig. 3:**
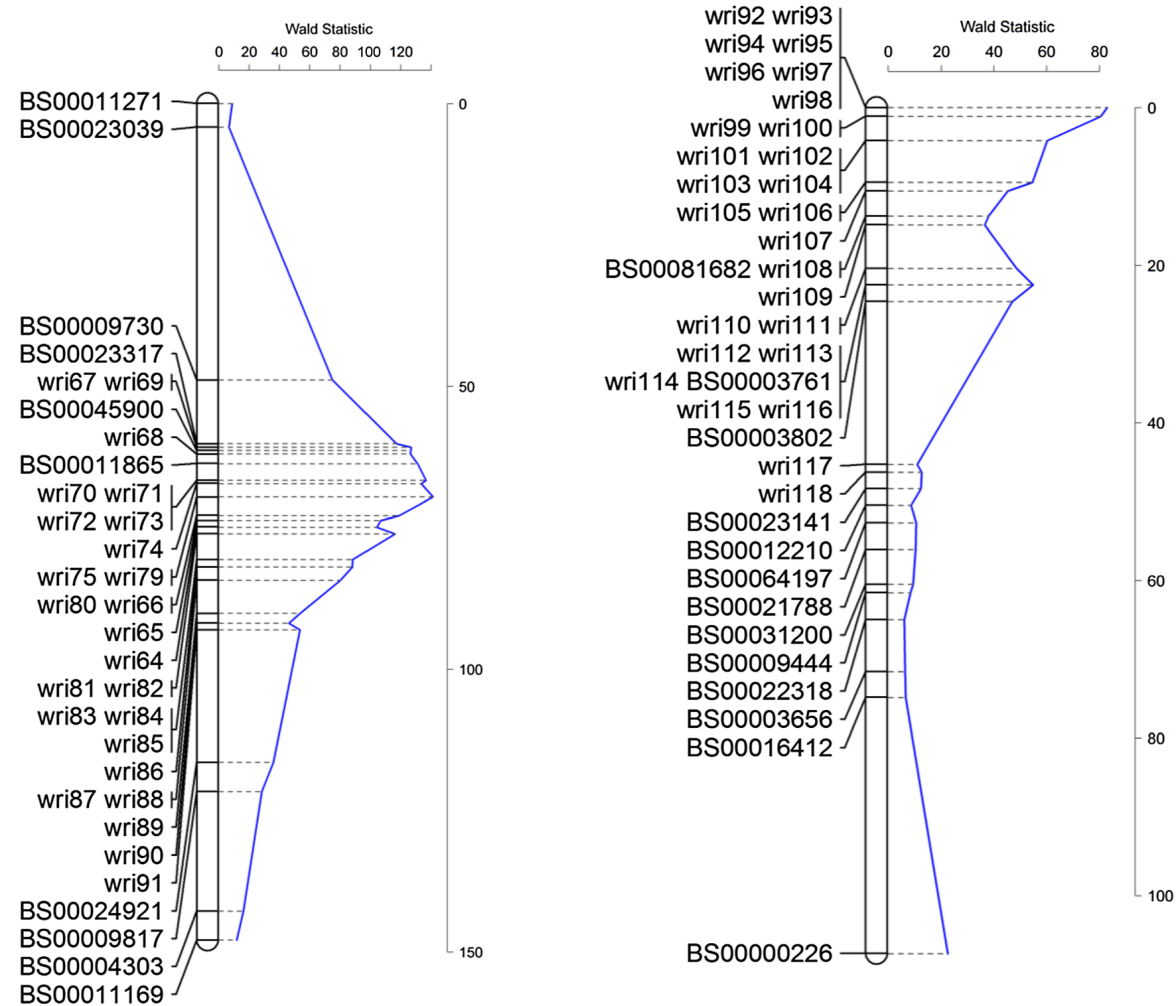
Multi-experiment tan spot resistance QTL Wald statistic profile of chromosome 2A for Calingiri/Wyalkatchem (left) and chromosome 1A for IGW2574/Annuello (right)

The ME QTL Wald statistic profile for a SNP-based linkage map of chromosome 2A had a highly significant peak at near markers *wri75* and *wri79* (Fig. 3). These markers were significantly associated (LOD > 3) with tan spot severity in all experiments except at the tillering stage of the glasshouse experiment conducted at South Perth in 2010 and the adult stage of the controlled environment experiment conducted at Toowoomba in 2013 (Table 4). From a baseline of 50% tan spot severity the presence of the favourable allele at the QTL reduced disease severity by 9–14% in field conditions (Table 4).

### QTL analysis: IGW2574/Annuello

In the IGW2574/Annuello population, a major QTL was detected on chromosome 1A in multiple experiments, with the Annuello parental allele favouring resistance (Table 3). The only other tan spot QTL with effects in more than one environment are on chromosomes 2D (Horsham 2010 and South Perth 2011) and 4A (South Perth 2009 and 2011). In the experiment conducted in South Perth in 2009, there was also a QTL on chromosome 5B, with a major effect on tan spot severity on adult plants. However, this QTL was similar in location to a QTL with major effects on plant development (Zadoks growth stage) in both 2009 and 2010, indicating that this disease resistance QTL may be an artefact of differential developmental rates of the lines.

**Table 3:** Quantitative trait loci detected in the IGW2574/Annuello population

Chromosome (linkage group)	Flanking markers (cM positions)	Trait	Location	Year	Environment type	*p*	% Variance	LOD	Source of allele that reduces trait value
1A	*wPt*-*2527* (4.3)–*wPt*-*744902* (5.3)	Tan spot severity (tillering)	Horsham	2010	Field	0.000	25.4	8.9	Annuello
		Tan spot severity (adult)	South Perth	2009	Field	0.000	9.1	2.6	Annuello
		Tan spot severity (adult)	South Perth	2011	Controlled	0.000	12.8	3.1	Annuello
		Tan spot severity (adult)	Toowoomba	2013	Controlled	0.000	28.1	3.6	Annuello
	*wPt*-*1418* (32.5)–*wPt*-*667155* (37.2)	Tan spot severity (tillering)	Toowoomba	2011	Field	0.000	16.6	4.7	Annuello
1B	*wPt*-*5347* (13.5)–*wPt*-*6592* (14.8)	Tan spot severity (booting)	Horsham	2010	Field	0.001	9.0	2.2	IGW2574
2B	*wPt*-*733929* (162.7)–*tPt*-*9767* (165.8)	Tan spot severity (tillering)	Horsham	2010	Field	0.000	9.6	3.5	IGW2574
2D (2)	*wPt*-*666656* (21.4)–*wPt*-*3728* (27.6)	Tan spot severity (adult)	South Perth	2011	Controlled	0.002	6.8	1.7	Annuello
	*wPt*-*668239* (38.8)–*wPt*-*3692* (43.9)	Tan spot severity (tillering)	Horsham	2010	Field	0.000	19.7	7.3	Annuello
4A (1)	*wPt*-*7612* (1.1)–*wPt*-*9445* (14.8)	Tan spot severity (adult)	South Perth	2011	Controlled	0.003	6.0	1.6	IGW2574
	*wPt*-*9445* (14.8)–*wPt*-*7524* (17.5)	Tan spot severity (adult)	South Perth	2009	Field	0.000	12.9	4.1	IGW2574
4B (2)	*wPt*-*5559* (7.3)–*wPt*-*731635* (20.1)	Tan spot severity (tillering)	Horsham	2010	Field	0.000	8.0	3.0	Annuello
5B (2)	*wPt*-*3049* (0.0)–*wPt*-*5896* (16.4)	Tan spot severity (adult)	South Perth	2009	Field	0.000	21.0	6.5	IGW2574
7A	*wPt*-*2266* (0)–*wPt*-*3393* (27.8)	Tan spot severity (booting)	Horsham	2010	Field	0.005	6.8	1.4	Annuello
	*wPt*-*744897* (112.3)–*wPt*-*7122* (114.0)	Tan spot severity (booting)	Horsham	2010	Field	0.000	13.7	2.8	Annuello
7B	*wPt*-*0635* (126.2)–*wPt*-*2356* (136.7)	Tan spot severity (booting)	Horsham	2010	Field	0.003	12.2	1.6	IGW2574
	*wPt*-*3190* (155.4)–*wPt*-*1957* (156.4)	Tan spot severity (booting)	Horsham	2010	Field	0.004	10.3	1.5	IGW2574
	*wPt*-*7720* (157.0)–*wPt*-*8561* (159.6)	Tan spot severity (tillering)	Horsham	2010	Field	0.000	18.6	7.2	IGW2574
7D	*wPt*-*744843* (0)–*wPt*-*744219* (1.03)	Tan spot severity (booting)	Horsham	2010	Field	0.001	9.4	2.2	IGW2574

The ME Wald statistic profile indicated a highly significant QTL linked to a set of co-locating KASP markers on the short arm of chromosome 1A (Fig. 3). The marker(s) at this location were significantly associated (LOD > 3) with tan spot severity as assessed at the adult stage in the field at South Perth in 2010 and the controlled environment in Toowoomba in 2013, at tillering in the field at Horsham in 2010 and at Toowoomba in 2011 (Table 4). When the favourable allele was present disease severity was reduced by 7–15% in field conditions (Table 4).

**Table 4:** Logarithm base 10 of odds (LOD) score and average reduction (Red) in the percentage of tan spot severity achievable for the marker(s) linked to the putative QTL given in Fig. 3

Location	Year	Environment type	Trait	Calingiri/Wyalkatchem		IGW2574/Annuello	
				LOD	Red	LOD	Red
South Perth	2009	Glasshouse	Tan spot severity (seedling)	5.04	7.62	0.03	1.38
		Field	Tan spot severity (tillering)	4.93	11.53	0.94	7.33
			Tan spot severity (adult)	7.58	14.08	2.45	11.65
	2010	Glasshouse	Tan spot severity (seedling)	6.20	7.37	0.75	3.61
			Tan spot severity (tillering)	2.04	5.48	1.23	4.63
		Field	Tan spot severity (adult)	7.39	10.98	3.83	14.56
	2011	Controlled	Tan spot severity (seedling)	–	–	1.98	7.46
			Tan spot severity (adult)	–	–	3.11	12.65
Horsham	2010	Glasshouse	Tan spot severity (seedling)	11.80	9.43	0.71	3.92
		Field	Tan spot severity (tillering)	12.39	12.53	7.54	10.98
			Tan spot severity (booting)	7.01	8.39	2.15	5.58
Toowoomba	2011	Field	Tan spot severity (tillering)	7.09	8.50	6.70	10.93
	2012	Controlled	Tan spot severity (seedling)	3.27	11.69	0.27	2.16
	2013	Controlled	Tan spot severity (seedling)	17.57	14.06	0.25	2.62
			Tan spot severity (adult)	0.63	3.38	4.27	10.23

Reductions are based on a fixed level of 50% tan spot severity

### Evaluation of lines with resistance alleles at three loci

**Fig. 4:**
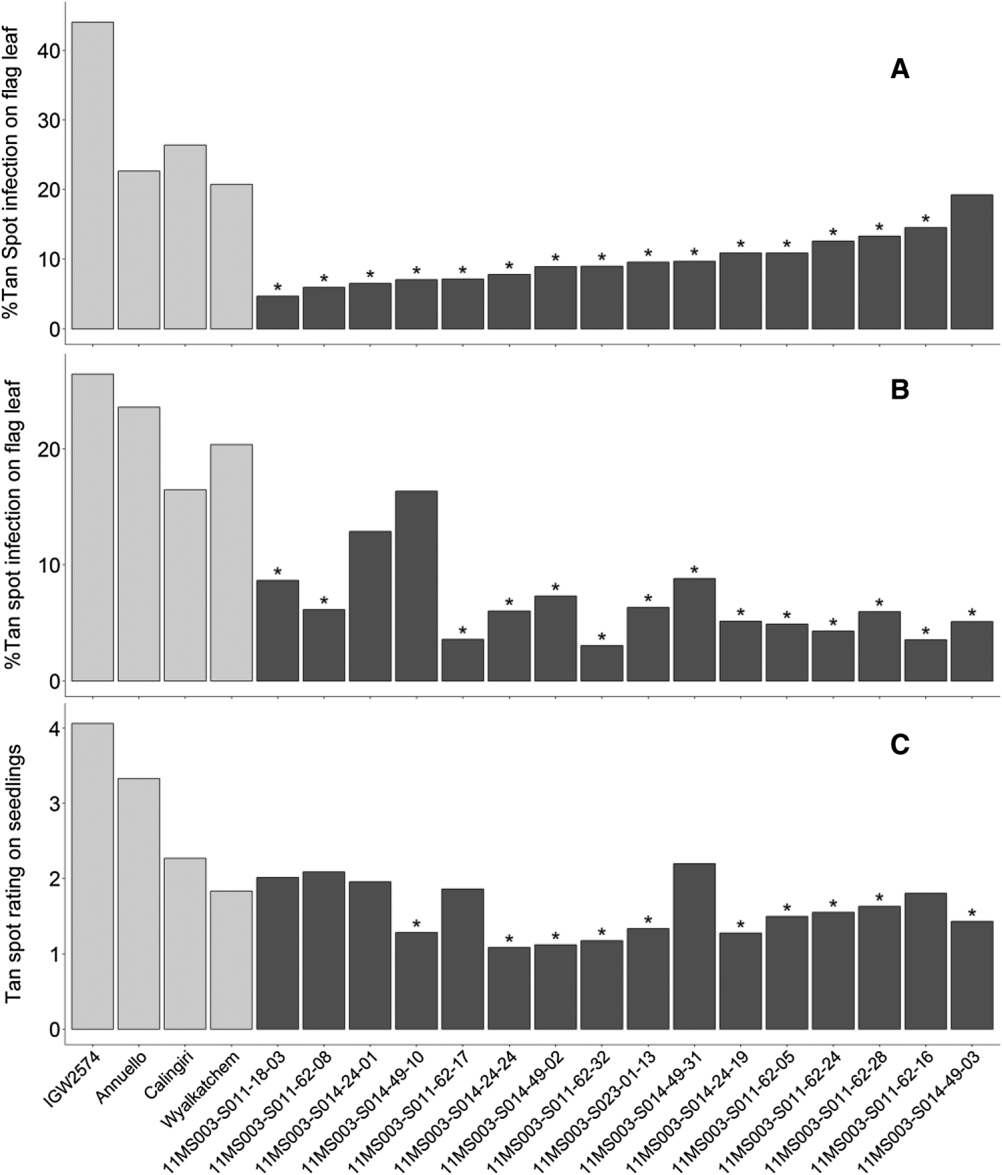
Comparison of 16 derived F_5_ lines (black bars) with stacked tan spot resistance loci (1A, 2A and *tsn1*) and the original parents of the DH populations (grey bars). **a** Back-transformed BLUEs of percentage flag leaf tan spot severity measured at the adult stage in the field experiment; **b** back-transformed BLUEs of the percentage flag leaf tan spot severity measured at the adult stage in the controlled environment experiment; **c** BLUEs of the tan spot severity rating measured at the seedling stage of the controlled environment experiment. “*” indicates F_5_ lines exhibiting significantly lower tan spot severity than Calingiri (which has only *tsn1*)

For the 16 selected F_5_ lines and the parents of the two DH populations, back-transformed BLUEs of the percentage flag leaf tan spot severity estimated at the adult stage in field and controlled environment experiments and BLUEs of the tan spot severity rating assessed at the seedling stage in controlled environment are presented in Fig. 4. For tan spot severity measured on the flag leaf at the adult stage in the field, 15 of the 16 F_5_ lines with the three combined resistance loci (*tsn1*, 1A and 2A) exhibited significantly (*p* < 0.05) reduced severity than Calingiri (which has *tsn1* alone) and Annuello (which has 1A alone) while 14 lines exhibited significantly reduced severity than Wyalkatchem (which has *tsn1* and 2A). All 16 lines were significantly better than IGW2574 which has no resistance alleles. For tan spot severity measured at the adult stage in the controlled environment 14 lines exhibited significantly reduced severity than both Calingiri and Wyalkatchem while 15 lines exhibited significantly reduced severity than Annuello. All 16 lines were significantly better than IGW2574. At the seedling stage, ten lines exhibited significantly reduced severity than Calingiri and three lines significantly reduced severity than Wyalkatchem. All 16 lines were significantly better than both Annuello and IGW2574.

## Discussion

Consistent with the expectation that resistance against tan spot in *tsn1* × *tsn1* and *Tsn1* × *Tsn1* crosses might be under polygenic control, the genetic predictions of the DH lines exhibited continuous distributions and transgressive segregation.

The high heritability estimates obtained for tan spot severity in most trials and strong genetic correlations of tan spot severity between environments indicate that disease expression was consistent and that the phenotyping methods used were reliable and precise. Considering the number of experimental variables between the three regions, such as, isolates used, temperature and rainfall patterns and inoculation and rating protocols, it was not surprising that a few experiments showed weak genetic correlations. Only race 1 of the pathogen is recorded in Australia (Antoni et al. 2010) and as hypothesised by Keller et al. (1997) population differentiation is not expected to be strong in a system of quantitative resistance as compared to systems with qualitative resistance. The resistance loci identified in this material should, therefore, be relevant and applicable to the breadth of Australian wheat production environments. Nevertheless, it is important to continue to monitor the pathogen to understand its full extent of variation across Australia and to determine its potential for pathotype co-evolution associated with resistance gene deployment.

The data from South Perth field trials indicated that while plant height and maturity could have had confounding effects on disease severity in 2009, these effects were successfully removed in 2010 and 2011 by modification of experimental protocols. This included inoculating individual plots at a specific growth stage and rating at a specific thermal time after inoculation. The strong correlation between assessments under controlled environment and field conditions at South Perth indicated that it is possible to phenotype materials all year round in controlled environments using methods that are faster and easier than those used in the field. Under controlled environment conditions of extended photoperiod and augmented fertilisation, plants showed rapid development producing heads and robust flag leaves within 5–8 weeks as compared to 11–16 weeks in the field. Well-developed tan spot symptoms were obtained on the flag leaves under these conditions and various resistant and susceptible lines were well distinguished. This method also allows for assessment at both seedling and adult stages on the same plant. Furthermore, enhanced spore production (80,000–130,000 spores per Petri plate) using the modified method of Evans et al. (1993) at South Perth and the ability to store sporulating plates at −20 °C for up to 1 year facilitated precise, quantifiable and repeatable studies.

Of the QTL detected here, those on chromosomes 2A (in Calingiri/Wyalkatchem) and 1A (in IGS2574/Annuello) were consistently detected across environments and growth stages. The QTL on 2A explained up to 29.2% genotypic variation while the QTL on 1A explained up to 28.1%. These loci may be the same as those previously reported by Faris et al. (1997, 1999), Effertz et al. (2001, 2002), Friesen and Faris (2004), Chu et al. (2008) and Li et al. (2011). KASP markers developed for these QTL will be crucial in breeding for resistance.

The continuous distributions observed for tan spot severity in the two mapping populations were similar to results obtained in other populations (Faris and Friesen 2005; Chu et al. 2010; Sun et al. 2010; Gurung et al. 2011; Li et al. 2011; Faris et al. 2012; Singh et al. 2012; and Patel et al. 2013) and may indicate that resistance is affected by multiple loci. Consistent with this, a number of QTL for tan spot severity traits, detected as significant in certain environments, occurred on seven chromosomes in the Calingiri/Wyalkatchem population and on 10 chromosomes in the IGW2574/Annuello population. These are of lesser interest for wheat breeding due to their minor effects and inconsistency across environments. Nevertheless, they demonstrate the complexity of resistance and its interaction with environmental conditions. Some of these QTL may correspond with previously reported QTL including loci on 2B (Li et al. 2011), 2D (Gurung et al. 2011; Patel et al. 2013); 3A (Chu et al. 2010; Singh et al. 2012); 4A (Chu et al. 2008); 4B (Singh et al. 2012) and 7B (Chu et al. 2010; Faris et al. 2012), but the QTL on 6B appears to be novel.

The resistance alleles of the QTL on 2A and 1A were successfully combined with resistance gene *tsn1* into fixed lines using single seed descent and marker-assisted selection. Most of the fixed lines with stacked resistance genes expressed significantly higher resistance than Calingiri (which has *tsn1* alone), Wyalkatchem (which has *tsn1* and 2A) and Annuello (which has 1A alone) at the adult plant stages that was effective in both the controlled environment and field. This higher resistance expression was less pronounced at the seedling stage, especially compared to Wyalkatchem, indicating that the QTL on 1A is more effective at the adult plant stage. These lines are important resources that can be used by breeders for rapid development of varieties with improved resistance. Research is currently underway to understand the effects of single tan spot resistance genes and genes in various combinations within isogenic lines.

### **Author contribution statement**

Conceived the experiments: MS, DEM, KJC and RL. Designed the experiments: MS, DEM, DJ, GJH and SMN. Performed the experiments: DJ, MS, RF, GJH, SMN, MSM, HG and EV. Analysed the data: KJC, RF, JT and MS. Wrote the manuscript: MS, DEM, JT, GJH and SMN.

## Electronic supplementary material

Below is the link to the electronic supplementary material.

Supplementary material 1 (XLSX 41 kb)

Supplementary material 2 (XLSX 25 kb)

Supplementary material 3 (XLSX 13 kb)
